# Treatment of Fecal Fistula

**Published:** 1897-02

**Authors:** 


### TREATMENT OF FECAL FISTULA.

WIGGIN, (*Med. Record,* October 24, 1896,) in a paper on above subject, said in case this mishap occurred and the opening was small with other favoring conditions operative measures might be postponed hoping the opening might heal spontaneously. If, however, the opening was large, above ileo-cecal valve, and large proportion of bowel contents escaped, operation was imperative. The histories of three cases were detailed. The operator laid stress upon (*a*) thorough disinfection of the parts, including interior of the bowel, with hydrozone; (*b*) closing intestinal opening when possible before breaking up peritoneal adhesions and opening the general cavity ; (*c*) removal of any existing obstruction to the fecal current ; (*d*) disinfection of the bowel surface with hydrozone before and after placing the sutures ; (*e*) control of oozing from cicatricial tissue by same means ; and (*f*) closure by single row of silkworm-gut sutures without drainage of abdominal wound, after washing the peritoneal cavity with saline solution, some of which is allowed to remain.

Wiggin stated that he proved the value of hydrogen dioxide in 1893 as an effective antiseptic, which in proper solution does not irritate the peritoneum.

